# A163 DIETARY PATTERNS OF BREASTFEEDING POST-PARTUM MOTHERS WITH INFLAMMATORY BOWEL DISEASE AND THEIR ASSOCIATIONS ON INFANT GROWTH

**DOI:** 10.1093/jcag/gwae059.163

**Published:** 2025-02-10

**Authors:** R Dang, R Y Wu, K O’Connor, A Epstein-Shapiro, K Xiao, V Srikanth, F Maiocco, V Huang

**Affiliations:** McMaster University, Hamilton, ON, Canada; University of Toronto, Toronto, ON, Canada; Sinai Health, Toronto, ON, Canada; University of Toronto, Toronto, ON, Canada; University of Toronto, Toronto, ON, Canada; Sinai Health, Toronto, ON, Canada; Sinai Health, Toronto, ON, Canada; Sinai Health, Toronto, ON, Canada

## Abstract

**Background:**

Inflammatory bowel disease (IBD), which includes Crohn’s disease (CD) and ulcerative colitis (UC) is characterized by chronic intestinal inflammation. Since IBD is often diagnosed in young adulthood, many people will have IBD at the time of pregnancy and breastfeeding. Chronic inflammation can cause severe nutritional deficiencies; however, it is unclear whether nutrient deficiencies during postpartum affect patients with IBD.

**Aims:**

To investigate the dietary patterns of breastfeeding mothers with IBD and correlate their dietary patterns with their infant growth within the first 6 months.

**Methods:**

A total of 21 mothers with IBD (9 CD and 12 UC) were recruited from the Preconception and Pregnancy IBD clinic at Mount Sinai. Inclusion criteria include females between 18-45 years olds with a diagnosis of CD or UC, who are at least 24 weeks gestational age, and females with multi-fetal gestation, co-morbid intestinal disease, or autoimmune comorbidities were excluded. Mothers completed a 3-day food diary and clinical disease activity scores at 1-month (1M) and 3-months (3M) postpartum, and self-filled questionnaires related to infant growth over a 6-month follow-up. Food and dietary patterns were analyzed using a food analysis software called ESHA based on age, gender, weight and height. Infant growth was compared to the World Health Organization’s growth curve Z-scores.

**Results:**

About half (52% at 1M and 48% at 3M) of mothers with IBD met the protein recommendation. Over half (62% at 1M and 3M) met their total fat recommendation. However, most did not meet the carbohydrate (33% at 1M and 19% at 3M) and fiber (24% at 1M and 10% at 3M) recommendations. All infants included in the analysis were breastfed at 1M and 3M postpartum. Breastfed infants born to mothers who met their total fat recommendation had higher length measurements compared to those who did not at 6 months.

**Conclusions:**

Our study findings show that breastfeeding mothers with IBD may not meet their recommended carbohydrate and fiber dietary intake, and that infants breastfed by mothers who do not meet their macronutrient dietary intake and lengths in the lower percentile range. Future research will delineate the specific nutrient composition in mothers’ breastmilk.

**Funding Agencies:**

American College of Gastroenterology, University of Toronto, CAN-TAP-TALENT

